# Development and application of a weighted change score to evaluate interventions for vasomotor symptoms in patients with breast cancer using regression trees: a cohort study

**DOI:** 10.1007/s10549-024-07360-4

**Published:** 2024-05-19

**Authors:** Katherine Marie Cole, Sharon McGee, Mark Clemons, Michelle Liu, Fiona MacDonald, Lisa Vandermeer, Terry L. Ng, Gregory Pond, Khaled El Emam

**Affiliations:** 1 Department of Medicine, Division of Medical Oncology, University of Ottawa, Ottawa, ON Canada; 2 School of Epidemiology and Public Health, University of Ottawa, Ottawa, ON Canada; 3 Cancer Therapeutics Program, The Ottawa Hospital Research Institute, Ottawa, ON Canada; 4 The Ottawa Hospital Cancer Centre, Ottawa, ON Canada; 5 Department of Oncology, McMaster University, Hamilton, ON Canada; 6 Children’s Hospital of Eastern Ontario Research Institute, 401 Smyth Road, Ottawa, ON K1H 8L1 Canada

**Keywords:** Breast cancer, Hot flashes, Vasomotor symptoms, Machine learning, Regression trees

## Abstract

**Purpose:**

Vasomotor symptoms (VMS) are common among individuals with breast cancer (BC) and poorly managed symptoms are associated with reduced quality of life, treatment discontinuation, and poorer breast cancer outcomes. Direct comparisons among therapies are limited, as prior studies evaluating VMS interventions have utilized heterogeneous change measures which may not fully assess the perceived impact of change in VMS severity.

**Methods:**

We performed a prospective study where BC patients chose one of four categories of interventions to manage VMS. Change in VMS severity at 6 weeks was assessed using the validated Hot Flush Rating Scale (HFRS). A novel weighted change score integrating baseline symptom severity and directionality of change was computed to maximize the correlation between the change score and a perceived treatment effectiveness score. Variables influencing change in VMS severity were included in a regression tree to model factors influencing the weighted change score.

**Results:**

100 baseline and follow-up questionnaires assessing VMS were completed by 88 patients. Correlations between treatment effectiveness and VMS outcomes strengthened following adjustment for baseline symptoms. Patients with low VMS severity at baseline did not perceive change in treatment effectiveness. Intervention category was predictive of change in HFRS at 6 weeks.

**Conclusion:**

Baseline symptom severity and the directionality of change (improvement or deterioration of symptoms) influenced the perception of clinically meaningful change in VMS severity. Future interventional studies utilizing the weighted change score should target moderate-high baseline severity patients.

**Supplementary Information:**

The online version contains supplementary material available at 10.1007/s10549-024-07360-4.

## Introduction

Vasomotor symptoms (VMS), including hot flashes and nocturnal sweats, are common treatment-related symptoms among individuals with breast cancer [1, 2]. These symptoms are associated with reduced quality of life [3, 4] and treatment discontinuation, which can lead to poorer breast cancer outcomes [5–8] and increased health services utilization [9]. A multitude of interventions for managing VMS have been evaluated in the breast cancer population, including enhanced lifestyle modifications [10], complementary and alternative medications (CAM) [11, 12], prescription medications [13, 14], and endocrine therapy modification (ETM) [15]. Direct comparisons among therapies, however, are limited by the heterogeneity of outcomes utilized in clinical trials, differences in the method/duration of measuring changes in outcomes, and limited comparisons of active therapies [16].

An important consideration when assessing the effectiveness of therapies is the clinical impact of change in VMS severity after an intervention, typically represented as the minimal clinically important difference (MCID). While the MCID is a commonly reported measure in VMS intervention trials, it is not monotonic, and there are several statistical and anchor-based approaches available to determine minimal change [17]. These heterogeneous approaches can lead to large variability in reporting and interpreting the MCID, making comparisons between active therapies difficult [16]. Additional complexity is compounded by wide diversity of VMS assessment tools, which capture variable symptom domains [16, 18]. Lastly, studies from the pain literature demonstrate that the perception of clinically meaningful change is influenced both by baseline symptom severity and the direction of change [19–24] (Supplementary Information 1). Extrapolating from these data, we hypothesized that similar findings may be applied to patients with breast cancer experiencing VMS.

**Fig. 1:**
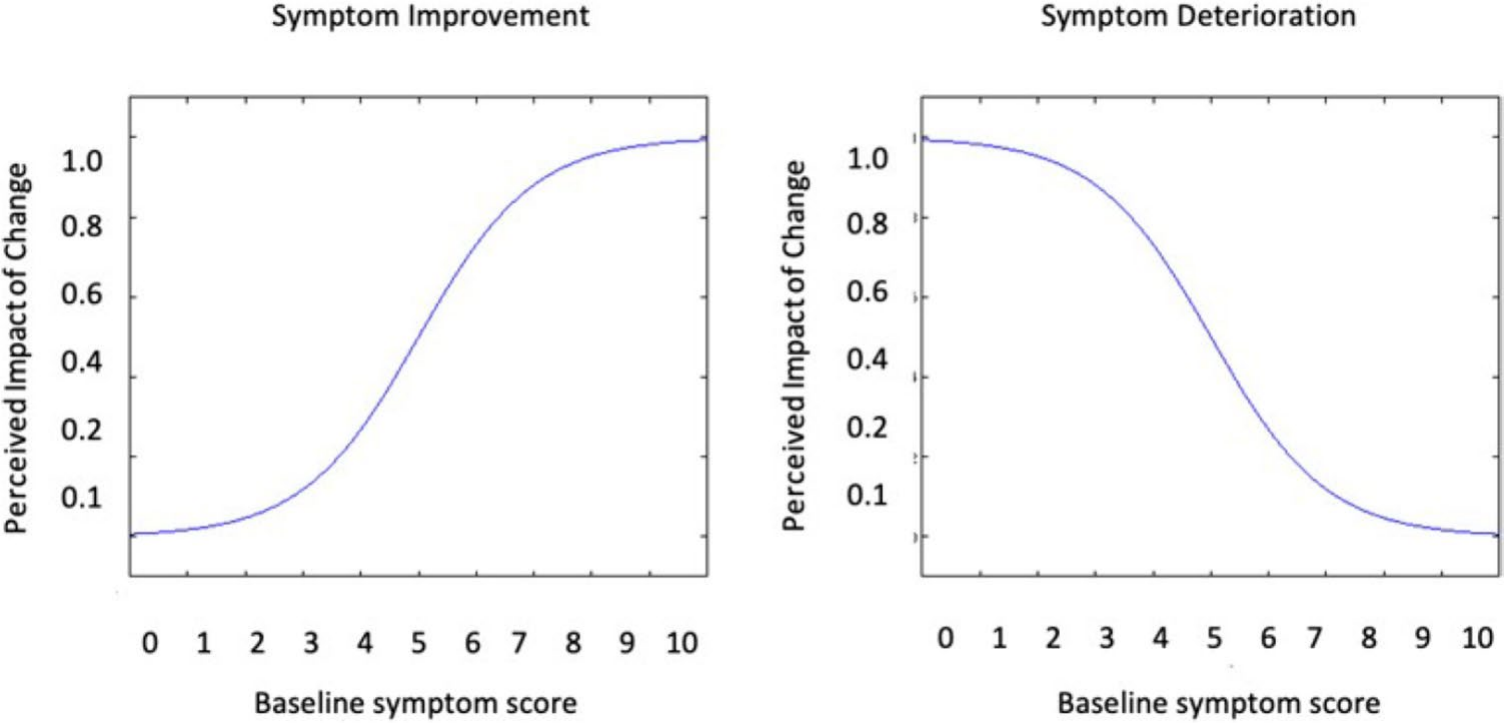
Hypothesized relationship between baseline symptoms and perceived impact of change among individuals experiencing an improvement (left panel) or deterioration (right panel) in symptom severity over time. Figure produced in Microsoft PowerPoint 2021

As psychometric functions can model stimulus–response relationships found in nature [25], we hypothesized that clinically important negative change in VMS severity (i.e., improvement) would follow a sigmoid curve, where individuals with a low severity score at baseline (i.e., lower symptom burden) would experience a smaller perception of change in VMS, and higher baseline severity score individuals (i.e., higher symptom burden) would experience a greater perception of change as illustrated in Fig. 1 (left panel). The converse would be true for individuals experiencing a positive change in severity (i.e., symptom deterioration), where individuals with low severity score at baseline (i.e., lower symptom burden) would experience a larger perception of change in VMS, and higher baseline severity score individuals (i.e., higher symptom burden) would experience a smaller perception of change as illustrated in Fig. 1 (right panel)**.**

In this study we examined whether the perceived impact of change in VMS severity depends on the baseline score and directionality of change as per the modeled relationship in Fig. 1 and subsequently developed a weighted change score to reflect this dependence. We then analyzed prospective data among breast cancer patients experiencing VMS to evaluate the impact of commonly utilized interventions on change in VMS severity. To accomplish this aim, we utilized the weighted change score, and regression trees as a modeling tool.

## Materials and methods

### Patient recruitment

We collected data from breast cancer patients experiencing VMS treated at the Ottawa Hospital Cancer Center (Ontario, Canada). Patients experiencing VMS of any severity were invited to participate in the study. Participants were given the option to pursue an evidence-based VMS intervention from one of 4 categories (lifestyle interventions, CAM, prescription medications, and endocrine therapy modification). Patients who did not wish to pursue an intervention were also eligible to participate as a control group. Recruitment occurred between September 3, 2021 and July 22, 2022. A list of included interventions is in Supplementary Information 2.

### VMS severity outcome

The study utilized the Hot Flush Rating Score (HFRS) which is a composite score to assess VMS severity [26]. The score takes the mean of three hot flash severity domains rated on a 10-point scale, including distress from hot flashes, problems caused by hot flashes, and disruption to daily life from hot flashes. This short assessment tool has strong internal consistency compared to expanded VMS questionnaires such as the HFRDIS, which measures comparable domains, and is associated with health services utilization and quality of life [9]. Patients also responded to a 5 level Likert question asking participants to respond to whether they deemed their treatment to adequately control their VMS (“Strongly disagree,” “disagree,” “neutral,” “agree,” “strongly agree”). This will be termed the “treatment effectiveness” score.

### Study procedures

Patients utilized MyChart™, a patient-centered application linked to the Epic electronic medical record, or Microsoft Forms™, to assess the severity of their VMS symptoms at baseline and 6 weeks using the HFRS instrument. Paper-based options were also available, and survey questions contained a combination of text responses, and discrete numeric data. Survey questions utilized in the analysis can be found in Supplementary Information 3. Participants were sent a two-week reminder questionnaire at 8 weeks if they had not yet responded to the follow-up survey, and individuals were able to complete the study more than once if desiring to utilize additional interventions.

### Data preparation

Survey responses provided in text format were converted into categorical variables. Where possible, selected VMS interventions were adjudicated by the study research assistant, either through direct confirmation with participants or review of the EMR. The treatment effectiveness Likert score was converted to discrete numeric data where “strongly agree” was given a score of 5 and “strongly disagree” was given a score of 1. Individuals who indicated “not applicable” on the Likert scale were converted to “neutral” scores. Interventions for VMS were classified into one of the 4 categories of interventions.

Change scores were calculated for the VMS severity outcome. An improvement in VMS severity was defined as a negative change in the severity score at 6 weeks (change score = X_2_-X_1_, where X_2_ is the follow-up score, and X_1_ is the baseline score). Non-improvement in VMS severity was defined as a positive change or no change in scores at 6 weeks.

### Data analysis

Descriptive statistics summarizing baseline patient characteristics and outcome data were conducted for patients who provided complete data for the VMS severity outcome measure. Individuals with complete outcome data for the HFRS outcome were defined as those who provided a numeric response for each of the three severity domains. Summary statistics were conducted at the level of a survey couplet (one response including a baseline and follow-up questionnaire).

Spearman correlations were computed to assess the relationship between the treatment effectiveness scores and the VMS severity change score, with stratification by individuals experiencing positive and negative change scores, respectively. This is a form of establishing the predictive validity of an assessment instrument [27]. Individuals included in the analyses were those who provided complete data for the HFRS score and the treatment effectiveness score. To assess our hypothesis that the perception of change varies with baseline score and directionality of change, a weighted change score (C’) integrating baseline scores was computed using the following equation $C^{\prime} = w \times C$, where “C” is the raw change score and “w” is the weight accounting for baseline effect defined by the function

1 $$w = \frac{1}{{1 + e^{{ - \left( {b_{0} + b_{1} \times X_{1} } \right)}} }}$$

The optimal beta parameters (b0 and b1) were estimated utilizing Bayesian optimization to maximize the Spearman correlation between C’ and the treatment effectiveness score [28].

Clinical variables influencing change in VMS severity at 6 weeks were selected for inclusion in regression tree models [29] (see Supplementary Information 4). Unlike traditional regression methods, regression trees are capable of modeling multi-level interactions without strict statistical assumptions and a priori model specification [30]. Regression trees are robust in datasets with large amounts of missing data, as the algorithm is able to account for missing variables without explicit imputation [31]

Variable selection was based on clinical expertise and a literature review of known factors which are predictive of change in VMS, and included a combination of patient characteristics, the VMS intervention category, and time-relevant variables. Collinearity testing using the variance inflation factor was performed to avoid redundancy between selected independent variables. Data-driven methods, such as degree of missingness and univariate testing of significance, were not utilized to guide variable selection.

The included variables were time in days between completion of baseline and follow-up questionnaires (2 levels), VMS intervention (5 levels), age (3 levels), patient-reported adherence (in days) to chosen VMS intervention (continuous), menopausal status at time of breast cancer diagnosis (2 levels), and duration of endocrine therapy (in months) at time of baseline questionnaire completion (2 levels). Other variables influencing baseline severity, including type of systemic cancer therapy (endocrine therapy, chemotherapy) [32], were not included in the model, as these would be expected to influence baseline severity scores, which would already be adjusted for as part of the weighted change score.

Categorization of independent variables was based on biological considerations (e.g., menopausal status) or were selected based on previous research work [32]. While age and menopausal status were significantly associated on chi-square testing, endocrine therapy is a risk factor for treatment-associated menopause [1], and as such, both variables were included. Endocrine therapy was dichotomized based on published data indicating that VMS are most severe during the first 12 months on therapy [33]. Time-relevant variables (time to survey completion) were dichotomized to incorporate the 2-week email reminder period. Adherence to the intervention was left as a continuous variable. Missingness levels for the included variables are provided in Supplementary Information 4.

The outcomes used in the regression tree analyses was the weighted change scores defined as a continuous variable, which was derived from the optimal b0 and b1 parameters identified in the weighted change analysis. Ten-fold nested cross-validation was utilized for model training and internal validation. For the inner loop, five-fold cross-validation and Bayesian optimization were utilized to estimate the model hyperparameters. Boundaries for hyperparameters of interest included the following: minimum number of observations in the parent node (5–20), minimum number of observations in terminal node (2–10), complexity parameter (0.001–0.1), and maximum depth of the tree (3–7 levels). Model performance was computed as the mean-squared error (MSE). For the Bayesian optimization, twenty points were selected to sample the target function, with 10 repeats. An upper confidence-bound acquisition function was selected with a kappa of 2.576. For this type of analysis, nested cross-validation does not overestimate model accuracy [34, 35].

## Results

### Source data

One hundred and twenty seven individuals were recruited, completing 147 baseline questionnaires. A total of 104 follow-up questionnaires were completed by 88 unique patients, with 39 individuals lost to follow-up. Eighty-five individuals provided complete outcome data from 100 questionnaires for the HFRS outcome.

### Baseline characteristics

Baseline characteristics for study participants are displayed in Table 1**.** The median age of participants was 52.0 (IQR 46.3–61.0 years). Eighty-two percent of respondents (*n* = 82) reported taking endocrine therapy at the time of study entry, and the median duration of time on current therapy was 9.9 months (IQR 4.0–20.9 months). Eighty-eight percent of respondents (*n* = 88) reported previously utilizing lifestyle interventions to manage their VMS, and forty-two percent (*n* = 42) and twenty-one percent (*n* = 21) reported previously utilizing non-lifestyle interventions (CAM, prescription drugs) and endocrine therapy modifications, respectively. All participants excluding one individual had localized breast cancer and 2 individuals chose to complete the survey on paper. There were no significant differences in baseline characteristics between participants who completed at least one follow-up questionnaire and those who completed the baseline questionnaire only (Table 1).

**Table 1:** Baseline Participant Characteristics

Patient Characteristics	Study Population (*n* = 100) *	Baseline only (*n* = 147)	*p* value^**¶¶**^
Age (years), median(IQR)	52.0 (46.3, 61.0)	54.0 (47.0, 62.0)	0.10
Pre/perimenopausal status at BC diagnosis n (%)	51 (51)	68 (46)	0.08
Baseline HFRS Median (IQR)	5.0 (3.3–7.0)	5.0 (3.3, 6.7)	0.30
Vasomotor Symptoms per week Median (IQR)	38.0 (17.0–61.0)	32.5 (14.0, 58.8)	0.17
Current endocrine therapy (*n*%)	82 (82)	120 (82)	1.0
Tamoxifen	65 (65)	94 (64)	
Aromatase Inhibitor	16 (16)	25(17)	
Ovarian Function Suppression	17 (17)	22 (15)	
Time on current endocrine therapy (months)(median IQR)	9.9 (4.0–20.9)	9.8 (4.18, 20.9)	0.93
Previously utilized interventions† (*n*%)			
Lifestyle	88 (88)	129 (88)	0.90
CAM/prescription medications	42 (42)	63 (43)	1.0
Endocrine modification	21 (21)	30 (20)	0.97
Study Characteristics			
Median time to completion of follow-up questionnaire (IQR)	54.5 days (45.0 − 63.0 days)	NA**	NA
Median time on intervention (IQR)	50.0 (41.0, 60.0)	50.0 (42.0, 62.0)	0.50

^*^Indicates number of completed follow-up questionnaires. Denominators for participant proportions are reported inclusive of non-responders

^**^NA = not applicable-Values reflect those who completed follow-up questionnaire only

^**¶**^All controls derived from study #1, comprising individuals who did not pursue an intervention

^**†**^Indicates interventions that were tried prior to study entry

^**¶¶**^^**−**^p value reflects statistical comparison between individuals who completed at least one follow-up questionnaire vs those who completed the baseline questionnaire only. Bonferroni correction for multiple testing utilized

Participants reported experiencing a median of 38.0 VMS per week (IQR 17.0–61.0). The median baseline HFRS score was 5.0 (IQR 3.3–7.0). Seventy-one percent of respondents (*n* = 71/100) reported trying a new intervention during the study period. Lifestyle interventions and CAM therapies were the most preferred interventions **(**Table 2**).**

**Table 2:** Vasomotor Symptom Interventions Utilized by Study Participants

Intervention category	Specific intervention (n,% of category)
Lifestyle *(*n* = 27)	Yoga/Relaxation 7 (26%) Dietary Changes 10 (37%) **Environmental modifications 15 (56%)** **Exercise 18 (67%)** Smoking cessation 4 (15%)
CAM** (*n* = 25)	**Black cohosh 12 (48%)** Melatonin 5 (20%) Acteane 4 (16%) Evening primrose oil 3 (12%) Vitamin E 1 (4%)
Prescription medications (*n* = 11)	**Venlafaxine 4 (36%)** **Oxybutynin 4 (36%)** Clonidine 1 (9%) Escitalopram 1 (9%) Pregabalin 1 (9%)
Endocrine therapy modification (*n* = 8)	Change of therapy 2 (25%) **Dose reduction 3 (38%)** **Short treatment break 3 (38%)**

^*^Multiple options possible

^**^CAM complementary and lifestyle interventions

Bold indicates most commonly selected intervention(s) of category

The median duration of time on the intervention was 50.0 days (IQR 42.0–60.0). Individuals who tried an intervention did not have significantly higher baseline severity scores as compared to those who declined an intervention (mean 5.4 vs 4.6, t(98), t = − 1.69, *p* = 0.09 for baseline HFRS score.

### Change scores adjusting for baseline effect

Seventy-three percent (*n* = 73/100) of patients indicated a numeric improvement in HFRS scores at 6 weeks. Optimal parameters for the weighted change scores were computed. After removal of missing data, 59 individuals (59%) provided both (1) complete outcome data for the HFRS score and (2) responded to the Likert treatment effectiveness question. Sixteen (27%) patients had a deterioration and 43 (73%) had an improvement in VMS severity on the HFRS score.

**Fig. 2:**
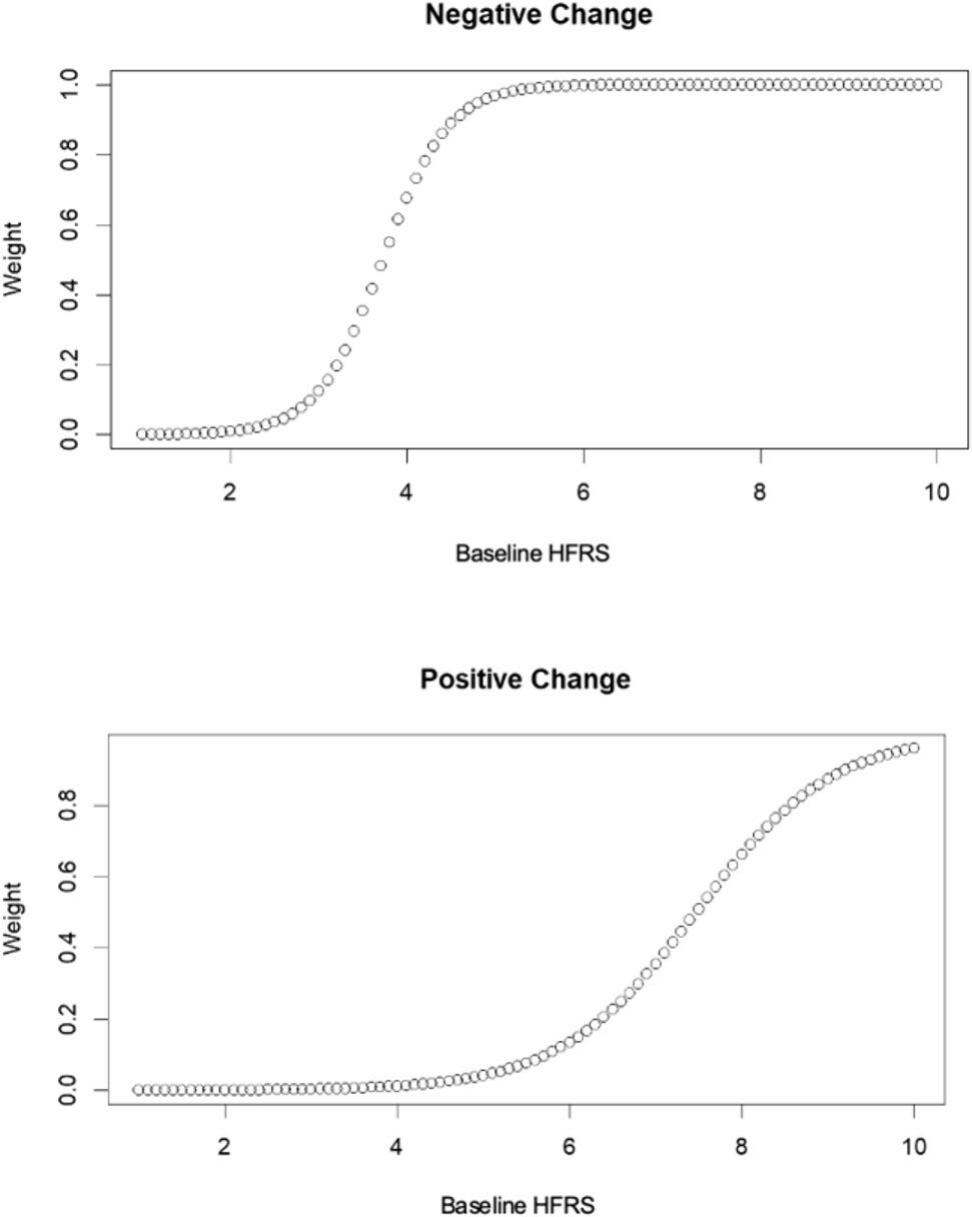
**a** (top pane) Baseline HFRS with associated weights among breast cancer patients with improvement in vasomotor symptoms at 6 weeks. **b** (bottom pane) Baseline HFRS with associated weights among breast cancer patients with deterioration in vasomotor symptoms at 6 weeks. Figure created in R(v 4.3.0) Following adjustment for the baseline effect, the median weighted change for the HFRS outcome was − 0.58 (IQR − 1.98– 0.00). The median change scores stratified by intervention are displayed in Supplementary Information 5

Individuals who reported an improvement in symptoms were more likely to report the intervention as effective (Spearman correlation = 0.15), whereas those reporting a deterioration in symptoms were less likely to report an intervention as effective (Spearman correlation = − 0.06); however, the correlations were weak. The correlations were not statistically significant (*p* = 0.82 for positive change, and 0.34 for negative change). Following optimization of the weighted change score integrating baseline VMS severity, the Spearman correlation among individuals with a negative change score (improved symptoms) was 0.27, and the correlation among individuals with a positive change score was − 0.20. The optimal parameters identified by the Bayesian optimization analysis for both positive change score were b0 = − 9.45 and b1 = 1.27, and for the negative change score were b0 = − 10 and b1 = 2.68. Among individuals experiencing an improvement or deterioration in symptoms, increasing baseline scores were associated with a greater weight (Fig. 2a and b).

### Regression trees for vasomotor symptom severity outcomes

**Fig. 3:**
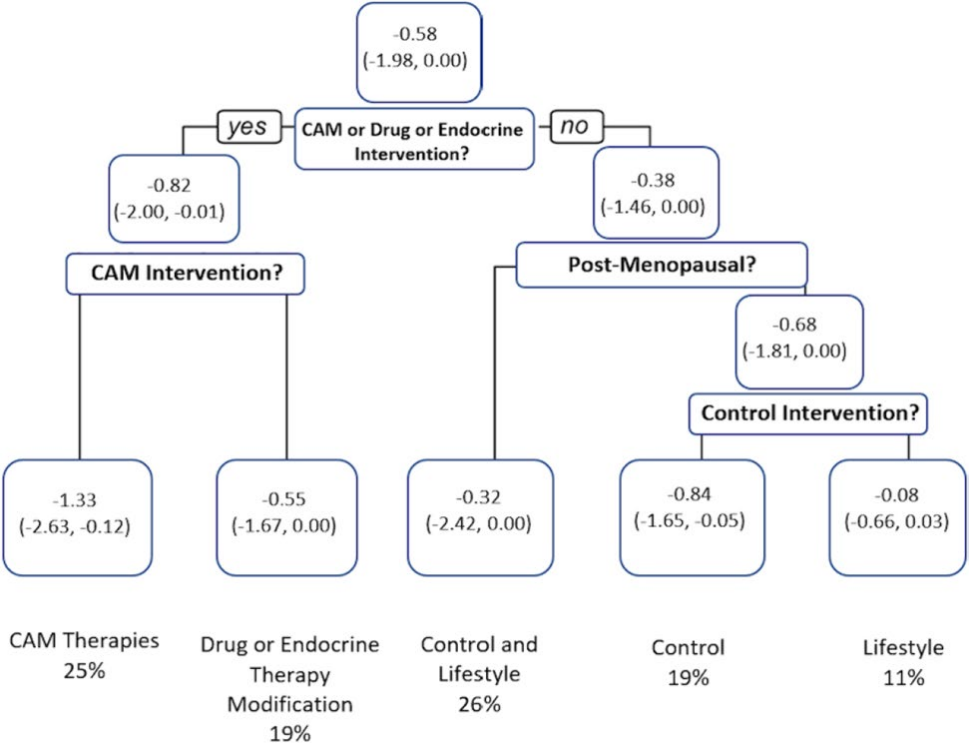
Regression tree for the impact of predictors on median change in HFRS severity at 6 weeks. Where question posed “yes,” values proceed to left, and “no” values proceed to right hand of tree. Values in terminal nodes represent the median and 95% confidence intervals. Figure created in R(v 4.3.0). *CAM* complementary and alternative medicine therapies, *LS* lifestyle, *Control* no new intervention, *Drug* prescription medications, *Endocrine modification* change to endocrine therapy

The optimal hyperparameters identified by ten-fold nested cross-validation and Bayesian optimization are indicated in Supplementary Information 6. The average MSE for the regression tree utilizing the weighted HFRS outcome was 1.77 (95% CI 1.85–1.96). The resulting trees highlighting the most important predictors for the outcomes of interest are demonstrated in Fig. 3**.**

Individuals utilizing CAM therapies reported the greatest numeric difference in HFRS change scores at 6 weeks (-median change score − 1.33, IQR − 2.63, − 0.12). Pre-menopausal women utilizing lifestyle interventions experienced the smallest reduction in severity scores (-median change score − 0.08, IQR − 0.66, 0.03).

Baseline differences between patients in select subgroups identified in the regression tree are displayed in Supplementary Information 7.

## Discussion

VMS symptoms are a significant problem among individuals with breast cancer, and optimal management remains a challenge. Recommendations on appropriate interventions are hampered due to multiple outcomes utilized in clinical trials, limited comparisons of active therapies [16, 36], and patient preferences for non-pharmacologic treatments. To facilitate accurate evaluation of interventions, we developed a weighted change score that accounts for baseline VMS severity, and for the directionality of VMS severity change after an intervention among breast cancer patients. This change score was optimized for predictive validity against perceived intervention effectiveness. We then applied these weighted change scores in regression trees to identify whether various interventions for VMS are predictive of an improvement in VMS severity at 6 weeks.

### Summary and key findings

To our knowledge, this is the first study which demonstrates that baseline symptom severity and the directionality of change (improvement or deterioration of symptoms) influence the perception of clinically meaningful change among patients with breast cancer experiencing VMS.

Our results demonstrate that while change becomes increasingly important at higher baseline severity scores regardless of the direction of change for the HFRS; the *threshold* at which change becomes meaningful depends on whether symptoms are improving or deteriorating. For those with symptomatic improvement, clinically important change was noted among individuals with moderate and high baseline scores, whereas important change was restricted to the highest severity score individuals for those experiencing a deterioration in symptoms. These findings suggest that patients are better able to tolerate an increase in symptom severity, whereas decreased severity (i.e., symptom improvement), is important across broader symptom spectrums. While these findings have not been explicitly studied among individuals experiencing VMS, the observation that meaningful perceived change varies depending on the directionality of change is supported by previous work conducted in the chronic pain population [23, 24].

We have also demonstrated that change scores integrating baseline effects and directionality of change can be applied in regression trees to identify predictors of improvement in VMS severity at 6 weeks among patients with breast cancer. Intervention category was predictive of change in VMS, with CAM therapies were associated with the largest numerical difference in VMS severity. The tree findings need to be interpreted with caution, however, as interventions within each intervention category were heterogeneous, reports of the effectiveness of these interventions are mixed [12, 37], the sample size is small, and the placebo effect can induce up to a 50% change in VMS scores in the breast cancer population [38].

### Application of results

As an application of our change score, we can consider two patients with baseline scores of “6” (Patient “A”), and “4” (“Patient B”). To reflect improvements typically reported in VMS intervention studies [39], both patients experience a 50% improvement in raw HFRS scores. Utilizing the curve from Fig. 2a and Eq. 1, a 50% (three-point) reduction in scores for patient “A” would be multiplied by a weight of “1”, resulting in an identical weighted change score of 3 points. For patient “B”, however, a 50% (two-point) reduction in raw HFRS scores would be multiplied by 0.6, resulting in a weighted change score of 1.2. Similarly, to experience a 50% improvement in HFRS scores accounting for baseline severity, Patient “B” would require a 3.3-point reduction in the raw HFRS score (2/0.6). Patient “B” has a lower baseline score, and thus requires a larger change in raw scores compared to Patient “A” to observe a comparable improvement. This example further highlights the limitations of fixed MCID thresholds when evaluating the importance of symptom change in the VMS population. Our results moreover suggest that individuals with moderate-high baseline severity scores should be selected for inclusion in future clinical studies, as these individuals are most likely to perceive change in VMS as meaningful and are thus expected to derive clinical benefit from VMS interventions.

### Limitations

For the weighted change analysis, nearly one-third of individuals declined an intervention, and as such, the sample size for the analysis was small. We created a limited number of trees for this study, and the small sample size increases the risk of overfitting of the dataset with under-estimation of the mean-squared error, although the nested cross-validation alleviates some of that concern. Moreover, certain factors such as race/ethnicity potentially influencing baseline severity were not available in the dataset; however, this would be adjusted for as part of the baseline score. The timing of initiation of chemotherapy and radiation therapy relative to endocrine therapy could potentially mitigate change in VMS, however, were not available for analysis.

Given the absence of an external population, we utilized the same dataset to perform the weighted change analysis, as well as the CART analysis, which can introduce biases in model performance. We moreover have not tested the predictive capabilities of our CART model in an external population of breast cancer patients experiencing VMS.

## Conclusion

We have outlined a novel technique to determine a weighted change score for VMS in breast cancer patients accounting for baseline severity. This approach has potential applicability across a broad range of patient-reported outcome measures and cancer disease sites.

### Reporting checklists

The TRIPOD checklist [40] is included with the supplementary materials (Supplementary Information 8). The consolidated JMIR AI checklist [41] is also completed (Supplementary Information 9) as the TRIPOD did not specifically cover AI modeling studies at time of submission.

### Supplementary Information

Below is the link to the electronic supplementary material.

Supplementary file 1: (PDF 498 KB)

## Data Availability

The analysis was performed using R statistical software (v 4.3.0). The R markdown file is available from Dr El Emam’s Electronic Health Information Laboratory website: (https://ehealthinformation.ca/blog/EHIL%20Blog/R%20Files). The dataset consists of personal health information and therefore would not be available for sharing in raw format. Requests for access to de-identified or synthetic variants should be made to the authors.

## Declarations

### Research involving human and animal participants

Patients were involved in the question development/piloting of earlier hot flashes surveys, and some of these questions were utilized in the current project.
