# Supplementary material for: Development and application of a weighted change score to evaluate interventions for vasomotor symptoms in patients with breast cancer using regression trees: a cohort study
Source: Breast Cancer Res Treat. 2024 May 19;207(2):313–21. doi: 10.1007/s10549-024-07360-4 (PMC11297085; doi:10.1007/s10549-024-07360-4)
Supplement: Supplementary file 1 — (PDF 498 KB) [file 10549_2024_7360_MOESM1_ESM.pdf]

Development and application of a weighted change score to evaluate interventions for vasomotor symptoms in patients with breast cancer using regression trees

Supplementary Materials

Authors:

Katherine Marie Cole[1,2], Sharon McGee[1,3], Mark Clemons[1,3], Michelle Liu[3], Fiona MacDonald[4], Lisa Vandermeer[3], Terry L. Ng[1,3], Gregory Pond[5], Khaled El Emam[2,6]

[1]Department of Medicine, Division of Medical Oncology, University of Ottawa, Ottawa, Ontario, Canada
[2]School of Epidemiology and Public Health, University of Ottawa, Ottawa, Ontario, Canada
[3]Cancer Therapeutics Program, The Ottawa Hospital Research Institute, Ottawa, Ontario, Canada
[4]The Ottawa Hospital Cancer Centre, Ottawa, Ontario, Canada
[5]Department of Oncology, McMaster University, Hamilton, Ontario, Canada
[6]Children's Hospital of Eastern Ontario Research Institute, Ontario, Canada

Corresponding author:

Dr Khaled El Emam, CHEO Research Institute, 401 Smyth Road, Ottawa, Ontario K1H 8L1, Canada; kelemam@ehealthinformation.ca; +1 613 737-7600

Keywords: breast cancer, hot flashes, vasomotor symptoms, machine learning, regression trees

Supplementary Information 1: Literature Review on Change Measurement

1.1. Vasomotor Symptoms in Breast Cancer Patients- Burden of the Problem

Vasomotor symptoms (VMS), inclusive of hot flashes and nocturnal sweats, are a common clinical concern among individuals with breast cancer [1,2]. While VMS are a major feature of natural menopause [3], a number of systemic therapies for breast cancer may also induce or exacerbate underlying VMS, including chemotherapy [4], ovarian function suppression [5] and endocrine therapies such as tamoxifen [6] and aromatase inhibitors [7]. Individuals with clinically bothersome symptoms have reduced quality of life, manifested by mood disturbances, cognitive changes, and impaired sexual health [8,9]**.** Moreover, VMS are associated with poorer adherence to endocrine therapy [10–12] resulting in reduced survival and increased risk of breast cancer recurrence [13].

A multitude of interventions for managing VMS have been evaluated in the breast cancer population, including enhanced lifestyle modifications [14] complementary and alternative therapies (CAM) [15,16], prescription medications [17,18], and endocrine therapy modification [19]. While estrogen-based therapies are typically utilized in the first line setting for managing VMS in the general population [20], these treatments are generally avoided in the majority of breast cancer patients due to estrogen's role in tumor development [21]. We therefore conducted a systematic review and network meta-analysis to evaluate the efficacy of non-hormonal interventions for VMS among breast cancer patients [22]. The review identified significant knowledge gaps, highlighting that direct comparisons between VMS interventions were limited by 1) differences in the patient reported outcome tools utilized for outcome assessment, and 2) differences in the methods and duration of change assessment across trials (for example, percent change from baseline vs mean change vs post- intervention scores) [22]. CAM and pharmacologic therapies were rarely directly compared against active treatments, while relationships between physical and psychological interventions could not be meta-analyzed given the absence of pairwise therapy comparisons to build the network [22].

In light of these findings, we then conducted two surveys of patients and health care professionals to better understand treatment and prescribing preferences in this population [23,24]. While the majority of patients expressed an interest in receiving an intervention for VMS, a minority of patients (5%) indicated a preference for prescription medications to manage their symptoms, with 61% indicating a specific preference for non-pharmacologic therapies [23]. This finding was in contrast to prescribing practices for health care professionals, where pharmacologic strategies were the most commonly recommended intervention after LS interventions in 74% of surveyed providers [24].

Severity of VMS among patients with breast cancer depends on several patient and treatment-specific factors. Patient-specific factors that increase the risk of VMS include modifiable (obesity, smoking) and non-modifiable (race/ethnicity) factors [25]. As mentioned above, among treatment-specific factors, chemotherapy, endocrine therapy, and ovarian function suppression can cause or exacerbate underlying VMS [4,5,26]. Despite these risk factors, baseline characteristics (eg. patient age, menopausal status) and treatment history (e.g. duration of time on endocrine therapy) are frequently under-reported in clinical trials evaluating therapies for VMS [22].

Given these knowledge gaps, we utilized the data from our previous cross-sectional patient surveys, and trained a gradient boosted decision tree to understand the predictors of VMS severity among breast cancer patients utilizing the validated hot flush rating score (HFRS) [27]. After including 17 patient and treatment-specific variables, we found that age and frequency of VMS were the most important predictors for perceived severity of VMS in this population [27].

In summary, while our previous work provided important perspectives on the efficacy of various interventions for VMS, we were unable to draw conclusions regarding change in symptoms over time, nor

longitudinally compare active therapies, given the cross-sectional nature of the study design. Moreover, given the retrospective nature of the survey questions, participant impressions of the effectiveness of interventions were subject to recall bias, thus limiting interpretation of results. Longitudinal surveys assessing symptoms over time are needed, however to effectively compare interventions, selection of an appropriate outcome for quantification of VMS is essential.

1.2. Outcome Selection for Measuring Response to VMS Interventions

There are several ways in which VMS outcomes are measured. Iliodromiti et al. broadly categorized VMS outcomes into 4 distinct domains: those evaluating the symptomatic experience of VMS (e.g. VMS frequency, duration, intensity), a domain exploring the impact of VMS on QOL, a domain exploring the functional impact/interference of VMS on daily activities, and finally, a domain evaluating VMS composite outcomes [28]. In contrast, our recent review conducted in breast and prostate cancer patients classified VMS into 3 domains: frequency, severity and composite score domains [22]. While these reviews demonstrate that classification of VMS outcomes can vary across trials, heterogeneity also exists *within* specific VMS domains. The review by Iliodromiti revealed up to 9 different definitions for VMS severity utilizing a variety of scales including visual analog scores, interval scores, and diaries evaluating frequency and/or symptom severity [28].

In addition to differences in the classification of VMS outcomes, clinical trials evaluating *change* in VMS outcomes vary in their approach. Common methods to measure change in VMS outcomes in the breast cancer population include: percent change from baseline (the most common technique), the absolute difference between baseline and follow up scores, and comparisons of follow-up scores between treatment groups [22] (Table 1). Finally, change in VMS outcome scores are measured across a variety of time points, with the majority of clinical trials evaluating change between 4 weeks and 12 weeks post treatment initiation [22]. Appropriately measuring change in symptom severity is essential to understanding the impact of an intervention on symptom reduction, however, there are several critical limitations for measuring change which will be further described below.

Table 1. Commonly Utilized Methods of Evaluating Change in Vasomotor Symptoms in Breast Cancer Patients

**Metric of Evaluating Change**	Formula	Strengths	Limitations
**Absolute difference**	X_2-X_1	Easy interpretation	Subject to RTM*
**% change from baseline**	$(X_2-X_1)/X_1 * 100\%$	Improves comparability of treatment groups	Subject to RTM*, introduces analytic complexity, altered data distribution due to normalization
**Comparison of follow-up scores**	Compare X_2 by treatment group	Easy interpretation, baseline adjustment less crucial if appropriate randomization	Subject to bias in non-randomized studies, or RCTs with small sample size

*RTM- regression to the mean, RCT= randomized controlled trial

1.3. Measuring Change

As discussed, change scores can be represented in several ways, and can include comparisons of post treatment scores, the absolute difference between pre-and post scores, the relative difference (e.g. taken as a percentage of the baseline) between pre and post test scores, and change scores standardized by the effect size [29,30]. Analysis of post-treatment scores are typically employed in the setting of a randomized controlled trial, where baseline factors are balanced between treatment groups. In the setting of small sample sizes, however, imbalances between treatment groups can emerge even in well-designed randomized studies [31].

Absolute and relative change scores, while simple to calculate and frequently utilized in VMS outcome trials [22], are subject to both regression to the mean (RTM) and floor/ceiling effects [29,32]. As an example highlighting RTM, an individual who reports low baseline symptoms by chance alone (e.g., a patient's baseline assessment is taken on a day when symptoms are atypically low) is more likely to drift towards the center of the scale on follow-up assessment. Regression to the mean becomes particularly important in studies where treatment groups have sufficiently different baseline features, leading to variability in the influence of RTM dependent on treatment allocation [29].

A comparable effect can be seen with floor and ceiling effects, however, these effects are reflected by limitations inherent to the scale itself [32]. As an example, consider an integer-based symptom scale where low numbers represent a state of "good health" or low symptom burden, and high numbers represent poor health or high symptom burden. Individuals with low baseline scores can improve by only a small amount as compared to individuals with moderate-high severity symptoms, as symptom improvement is truncated by the finite ends of the scale. Similarly, individuals with severe baseline symptoms can experience a finite deterioration in symptoms as compared to individuals with low-severity symptoms.

While relative change scores are highly interpretable by clinicians, this measure remains subject to RTM and thus under-performs compared to other analytic techniques such as analysis of covariance (ANCOVA) in the adjustment of participant baseline differences [33]. ANCOVA regresses the treatment group and a continuous post-treatment score with a baseline covariate adjustment [33]. While ANCOVA has superior statistical power in detecting treatment effect compared to absolute and relative change score analysis [33], it does assume comparable mean baseline scores between treatment groups, and its use should be avoided in studies where non-random differences in baseline scores would be expected [30]. Similarly, the power of relative change scores to detect statistical differences between treatment groups is poor when there is large variation in baseline values, [33,34].

Finally, relative change scores can over-estimate treatment response among individuals with lower symptom burden. For example, in order to experience a 50% change in symptom severity, an individual with a score of "2" on a 10 point severity scale need to experience a 1-point decrease in symptom severity to achieve this endpoint, whereas, an individual with a score of 10 needs to decrease their score by 5 points in order to experience a similar relative reduction. Due to unique properties of absolute and relative change scores, it has therefore been advocated by several guidelines to report both measures [35,36], however, as indicated above, both metrics can be influenced by RTM and floor/ceiling effects [32]. Regardless of the method selected, however, change score appraisal must be placed into clinical context, often reflected in the Minimal Clinically Important Difference (MCID).

1.4. The Minimal Clinically Important Difference- Defining Meaningful Change

The minimal clinically important difference (MCID) is utilized in clinical research as an outcome which attempts to define clinically meaningful change, and is frequently used to assess the efficacy of treatments for pain, chemotherapy induced nausea, and vasomotor symptoms, among others [37–39]. The MCID can be defined as the "smallest change in a patient reported outcome measure that is of genuine clinical

value" [40]. It is a metric that is rooted in patient perception [41], and aims to address the shortcomings of analyses evaluating strict statistical differences between interventions which may have negligible clinical value [32]. However, there are several limitations inherent in defining the MCID, which include both extrinsic and intrinsic factors [42].

Firstly, the MCID is dependent on the specific population of interest, and may vary depending on population age structures, comorbidities, baseline symptom severity, and length of follow up [32,41–43]. As such, identical changes in symptom scores may have varying clinical implications depending on the population of interest, thus limiting generalizability of this measure.

Secondly, there are 3 major methodologies for obtaining a minimal important difference (MID): anchor based methods, distribution based methods, and expert opinion/consensus methods [40]. Each of these has its own strengths and limitations (**Table 2**). The preferred methods of determining the minimal important difference, however, involves integration of clinical impressions/preferences, rather than purely statistical measures alone. Additionally, given the variability in these methods for determining the MID/MCID cross-study comparisons of trials utilizing these measures is challenging.

Table 2. Methods for Determining the Minimal Important Difference

	**Technique**	**Strengths**	**Limitations**
**Distribution**	Examines the distribution of an outcome using statistical measures including SD/SEM/ES/MDC* in a sample of interest	Straightforward analysis	Clinical impact of change/patient perception is not addressed
**Anchor-based**	Change in outcome measured against an external clinical anchor (e.g. global transition question)	Integrates patient perception of meaningful change	Results can vary dependent on choice of anchor, subject to outliers/concerns of internal reliability
**Receiver Operating Curve Analysis**	Identifies optimal threshold for clinically equivalent vs improved change scores by optimizing true positive rate and false positive rate	Integrates statistical and clinical based methods for determining meaningful change	Dependent on reliability of selected anchor

*SD= standard deviation, SEM= standard error of measurement, ES = effect size, MDC = minimal detectable change

Thirdly, there is a dependence between the MCID and the baseline score. Strict cut-offs for the MCID among intervention trials which do not account for baseline score can introduce mis-classification bias if participants have baseline values which are below or close to the threshold of the study defined MCID [44] . For example, if the absolute MCID is a 3-point difference, an individual with a baseline score of 2 would be incapable of "responding" to a specific intervention or treatment, irrespective of whether the

patient experiences subjective improvement in their symptoms. Conversely, as there is a positive correlation between higher baseline severity scores and raw change scores [38,41,45], among intervention studies which use strict MCID cut-offs, the proportion of individuals classified as deriving benefit from a therapy increases with increasing baseline score [44].

An additional consideration when evaluating the MCID for a specific disease and population, is whether the minimal clinical difference is the same for both individuals experiencing an improvement or deterioration in their symptoms. Several studies from the pain literature suggest that the amount of change to be considered meaningful is smaller among individuals who experience an improvement in symptoms, as compared to those who experience a deterioration in symptoms [46,47]. Several studies conducted in the orthopedics population have demonstrated that the MCID increases with increasing baseline severity, however, the patient acceptable symptom state (PASS), an outcome of overall perception of wellbeing, remains stable across severity tertiles [48]. The MCID, therefore, translates to the change needed to achieve the PASS. As such, individuals whose state of health is close to the PASS at baseline will likely require a smaller MCID as compared to those with high baseline symptoms who are farther from the PASS. A theoretical illustration of this is provided below in Figure 1, highlighting the stability of the PASS with gradual increase in MCID with baseline severity**.**

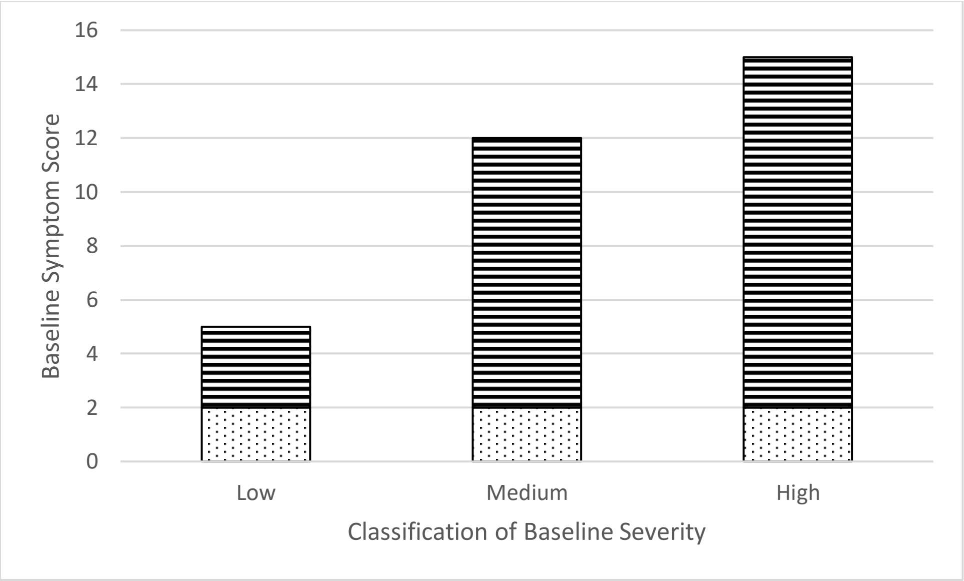

Figure 1. Relationship between Patient Acceptable Symptom State (PASS= dots) and Minimal Clinically Important Difference (MCID=lines)

There are mixed opinions regarding the best methods for integrating baseline scores when calculating the MCID. Concordance between different metrics of meaningful change (e.g. the MCID and the PASS) are improved when stratifying by baseline symptom score [49]. However, stratification of a study population into subgroups based on baseline score can introduce bias by redistributing measurement error [50].

In summary, appropriate comparisons between VMS interventions to date have been limited by a lack of comparisons between active therapies, heterogeneity of VMS outcomes and change measurements, and variable definitions of meaningful clinical change. Novel methods of evaluating and interpreting change

are needed to facilitate accurate comparisons between interventions, and to ensure more consistent measurement of VMS outcomes.

1.5. Weighting Change Scores by Baseline Symptom Severity

The influence of baseline symptoms on the perception of change among patients with VMS is poorly studied, and as such, we utilized data from the pain literature as a model for this concept. While distinct clinical entities, pain and VMS share many overlapping features in that they are both complex subjective experiences which include both physical and emotional domains. Data from the pain literature suggests that individuals with high baseline pain symptoms perceive the greatest symptom improvement from analgesic interventions [51–53], which has similarly been observed in the cancer population [54]. Moreover, among oncology patients with advanced disease, the introduction of early palliative care interventions for symptom management had the greatest impact among individuals with high vs low baseline symptoms [55]. Extrapolating from these data, we hypothesized that similar findings may be applied to breast cancer patients experiencing VMS. Modelling of the relationship between baseline symptoms and the impact of change is demonstrated in the main text.

Works Cited:

1 Howell A, Cuzick J, Baum M, *et al.* Results of the ATAC (Arimidex, Tamoxifen, Alone or in Combination) trial after completion of 5 years' adjuvant treatment for breast cancer. *Lancet Lond Engl* 2005;**365**:60–2. doi:10.1016/S0140-6736(04)17666-6

2 Paluch-Shimon S, Cardoso F, Partridge AH, *et al.* ESO-ESMO 4th International Consensus Guidelines for Breast Cancer in Young Women (BCY4). *Ann Oncol* 6;**31**:674–96. doi:10.1016/j.annonc.2020.03.284

3 Avis NE, Crawford SL, Green R. Vasomotor Symptoms Across the Menopause Transition: Differences Among Women. *Obstet Gynecol Clin North Am* 2018;**45**:629–40. doi:10.1016/j.ogc.2018.07.005

4 Son K-L, Jung D, Lee K-M, *et al.* Morning chronotype is a protective factor against chemotherapy-induced hot flashes in premenopausal women with breast cancer. *Support Care Cancer Off J Multinatl Assoc Support Care Cancer* 2020;**28**:1351–8. doi:10.1007/s00520-019-04949-0

5 Francis PA, Pagani O, Fleming GF, *et al.* Tailoring Adjuvant Endocrine Therapy for Premenopausal Breast Cancer. *N Engl J Med* 2018;**379**:122–37. doi:10.1056/NEJMoa1803164

6 Davies C, Pan H, Godwin J, *et al.* Long-term effects of continuing adjuvant tamoxifen to 10 years versus stopping at 5 years after diagnosis of oestrogen receptor-positive breast cancer: ATLAS, a randomised trial. *Lancet* 2013;**381**:805–16. doi:10.1016/S0140-6736(12)61963-1

7 Goss PE, Ingle JN, Pritchard KI, *et al.* Extending Aromatase-Inhibitor Adjuvant Therapy to 10 Years. *N Engl J Med* 2016;**375**:209–19. doi:10.1056/NEJMoa1604700

8 Utian WH. Psychosocial and socioeconomic burden of vasomotor symptoms in menopause: a comprehensive review. *Health Qual Life Outcomes* 2005;**3**:47. doi:10.1186/1477-7525-3-47

9 Woods NF, Mitchell ES. Symptoms during the perimenopause: prevalence, severity, trajectory, and significance in women's lives. *Am J Med* 2005;**118 Suppl 12B**:14–24. doi:10.1016/j.amjmed.2005.09.031

10 Yussof I, Mohd Tahir NA, Hatah E, *et al.* Factors influencing five-year adherence to adjuvant endocrine therapy in breast cancer patients: A systematic review. *The Breast* 2022;**62**:22–35. doi:10.1016/j.breast.2022.01.012

11 Fellowes D, Fallowfield LJ, Saunders CM, *et al.* Tolerability of hormone therapies for breast cancer: how informative are documented symptom profiles in medical notes for “well-tolerated” treatments? *Breast Cancer Res Treat* 2001;**66**:73–81. doi:10.1023/a:1010684903199

12 Barron TI, Connolly R, Bennett K, *et al.* Early discontinuation of tamoxifen: a lesson for oncologists. *Cancer* 2007;**109**:832–9. doi:10.1002/cncr.22485

13 Chirgwin JH, Giobbie-Hurder A, Coates AS, *et al.* Treatment Adherence and Its Impact on Disease-Free Survival in the Breast International Group 1-98 Trial of Tamoxifen and Letrozole, Alone and in Sequence. *J Clin Oncol* 7;**34**:2452–9. doi:10.1200/JCO.2015.63.8619

14 Efficacy of Cognitive Behavioral Therapy and Physical Exercise in Alleviating Treatment-Induced Menopausal Symptoms in Patients With Breast Cancer: Results of a Randomized, Controlled, Multicenter Trial. *J Clin Oncol*http://ascopubs.org/doi/pdf/10.1200/JCO.2012.41.8525 (accessed 2 Dec 2021).

15 Hernandez Torres C, Mazzarello S, Ng T, *et al.* Defining optimal control of chemotherapy-induced nausea and vomiting—based on patients’ experience. *Support Care Cancer* 2015;**23**:3341–59. doi:10.1007/s00520-015-2801-y

16 Lesi G, Razzini G, Musti MA, *et al.* Acupuncture as an Integrative Approach for the Treatment of Hot Flashes in Women With Breast Cancer: A Prospective Multicenter Randomized Controlled Trial (AcCliMaT). *J Clin Oncol* 5;**34**:1795–802. doi:10.1200/JCO.2015.63.2893

17 Pandya KJ, Morrow GR, Roscoe JA, *et al.* Gabapentin for hot flashes in 420 women with breast cancer: a randomised double-blind placebo-controlled trial. *Lancet Lond Engl* 2005;**366**:818–24. doi:10.1016/S0140-6736(05)67215-7

18 Buijs C, Mom CH, Willemse PHB, *et al.* Venlafaxine versus clonidine for the treatment of hot flashes in breast cancer patients: a double-blind, randomized cross-over study. *Breast Cancer Res Treat* 2009;**115**:573–80. doi:10.1007/s10549-008-0138-7

19 Jerusalem G, Farah S, Courtois A, *et al.* Continuous versus intermittent extended adjuvant letrozole for breast cancer: final results of randomized phase III SOLE (Study of Letrozole Extension) and SOLE Estrogen Substudy. *Ann Oncol* 2021;**32**:1256–66. doi:10.1016/j.annonc.2021.07.017

20 Yuksel N, Evaniuk D, Huang L, *et al.* Guideline No. 422a: Menopause: Vasomotor Symptoms, Prescription Therapeutic Agents, Complementary and Alternative Medicine, Nutrition, and Lifestyle. *J Obstet Gynaecol Can* 2021;**43**:1188-1204.e1. doi:10.1016/j.jogc.2021.08.003

21 Clemons M, Goss P. Estrogen and the risk of breast cancer. *N Engl J Med* 2001;**344**:276–85. doi:10.1056/NEJM200101253440407

22 Hutton B, Hersi M, Cheng W, *et al.* Comparing Interventions for Management of Hot Flashes in Patients With Breast and Prostate Cancer: A Systematic Review With Meta-Analyses. *Oncol Nurs Forum* 7;**47**:E86–106. doi:10.1188/20.ONF.E86-E106

23 Cole KM, Clemons M, Alzahrani M, *et al.* Vasomotor symptoms in early breast cancer-a "real world" exploration of the patient experience. *Support Care Cancer Off J Multinatl Assoc Support Care Cancer* 2022;**30**:4437–46. doi:10.1007/s00520-022-06848-3

24 Cole KM, Clemons M, Alzahrani M, *et al.* Developing patient-centred strategies to optimize the management of vasomotor symptoms in breast cancer patients: a survey of health care providers. *Breast Cancer Res Treat* 2021;**188**:343–50. doi:10.1007/s10549-021-06186-8

25 Thurston RC, Joffe H. Vasomotor symptoms and menopause: findings from the Study of Women's Health across the Nation. *Obstet Gynecol Clin North Am* 2011;**38**:489–501. doi:10.1016/j.ogc.2011.05.006

26 Morales L, Neven P, Timmerman D, *et al.* Acute effects of tamoxifen and third-generation aromatase inhibitors on menopausal symptoms of breast cancer patients. *Anticancer Drugs* 2004;**15**:753–60. doi:10.1097/00001813-200409000-00003

27 Cole KM, Clemons M, McGee S, *et al.* Using machine learning to predict individual patient toxicities from cancer treatments. *Support Care Cancer Off J Multinatl Assoc Support Care Cancer* Published Online First: 25 May 2022. doi:10.1007/s00520-022-07156-6

28 Iliodromiti S, Wang W, Lumsden M, *et al.* Variation in menopausal vasomotor symptoms outcomes in clinical trials: a systematic review. *BJOG Int J Obstet Gynaecol* 2020;**127**:320–33. doi:10.1111/1471-0528.15990

29 Angst F, Aeschlimann A, Angst J. The minimal clinically important difference raised the significance of outcome effects above the statistical level, with methodological implications for future studies. *J Clin Epidemiol* 2017;**82**:128–36. doi:10.1016/j.jclinepi.2016.11.016

30 Zhang S, Paul J, Nantha-Aree M, *et al.* Empirical comparison of four baseline covariate adjustment methods in analysis of continuous outcomes in randomized controlled trials. *Clin Epidemiol* 2014;**6**:227–35. doi:10.2147/CLEP.S56554

31 Wei L, Zhang J. Analysis of Data with Imbalance in the Baseline Outcome Variable for Randomized Clinical Trials. *Drug Inf J* 2001;**35**:1201–14. doi:10.1177/009286150103500417

32 Copay AG, Subach BR, Glassman SD, *et al.* Understanding the minimum clinically important difference: a review of concepts and methods. *Spine J Off J North Am Spine Soc* 2007;**7**:541–6. doi:10.1016/j.spinee.2007.01.008

33 Vickers AJ. The use of percentage change from baseline as an outcome in a controlled trial is statistically inefficient: a simulation study. *BMC Med Res Methodol* 2001;**1**:6. doi:10.1186/1471-2288-1-6

34 Tu Y-K. Testing the relation between percentage change and baseline value. *Sci Rep* 2016;**6**:23247. doi:10.1038/srep23247

35 Jp V, E von E, Dg A, *et al.* Strengthening the Reporting of Observational Studies in Epidemiology (STROBE): explanation and elaboration. *PLoS Med* 2007;**4**. doi:10.1371/journal.pmed.0040297

36 Kf S, Dg A, D M. CONSORT 2010 statement: updated guidelines for reporting parallel group randomized trials. *Ann Intern Med* 2010;**152**. doi:10.7326/0003-4819-152-11-201006010-00232

37 Mann E, Smith MJ, Hellier J, *et al.* Cognitive behavioural treatment for women who have menopausal symptoms after breast cancer treatment (MENOS 1): a randomised controlled trial. *Lancet Oncol* 2012;**13**:309–18. doi:10.1016/S1470-2045(11)70364-3

38 Olsen MF, Bjerre E, Hansen MD, *et al.* Minimum clinically important differences in chronic pain vary considerably by baseline pain and methodological factors: systematic review of empirical studies. *J Clin Epidemiol* 2018;**101**:87-106.e2. doi:10.1016/j.jclinepi.2018.05.007

39 Hendey GW, Donner NF, Fuller K. Clinically significant changes in nausea as measured on a visual analog scale. *Ann Emerg Med* 2005;**45**:77–81. doi:10.1016/j.annemergmed.2004.07.446

40 Bloom DA, Kaplan DJ, Mojica E, *et al.* The Minimal Clinically Important Difference: A Review of Clinical Significance. *Am J Sports Med* 2021;:3635465211053869. doi:10.1177/03635465211053869

41 McGlothlin AE, Lewis RJ. Minimal clinically important difference: defining what really matters to patients. *JAMA* 2014;**312**:1342–3. doi:10.1001/jama.2014.13128

42 Molino J, Harrington J, Racine-Avila J, *et al.* <p>Deconstructing the Minimum Clinically Important Difference (MCID)</p>. *Orthop Res Rev* 2022;**14**:35–42. doi:10.2147/ORR.S349268

43 Hays RD, Woolley JM. The concept of clinically meaningful difference in health-related quality-of-life research. How meaningful is it? *PharmacoEconomics* 2000;**18**:419–23. doi:10.2165/00019053-200018050-00001

44 Davis AM, Perruccio AV, Lohmander LS. Minimally clinically important improvement: all non-responders are not really non-responders an illustration from total knee replacement. *Osteoarthritis Cartilage* 2012;**20**:364–7. doi:10.1016/j.joca.2012.02.005

45 Olsen MF, Bjerre E, Hansen MD, *et al.* Pain relief that matters to patients: systematic review of empirical studies assessing the minimum clinically important difference in acute pain. *BMC Med* 2017;**15**:35. doi:10.1186/s12916-016-0775-3

46 Crosby RD, Kolotkin RL, Williams GR. An integrated method to determine meaningful changes in health-related quality of life. *J Clin Epidemiol* 2004;**57**:1153–60. doi:10.1016/j.jclinepi.2004.04.004

47 Cella D, Eton DT, Fairclough DL, *et al.* What is a clinically meaningful change on the Functional Assessment of Cancer Therapy-Lung (FACT-L) Questionnaire? Results from Eastern Cooperative Oncology Group (ECOG) Study 5592. *J Clin Epidemiol* 2002;**55**:285–95. doi:10.1016/s0895-4356(01)00477-2

48 Tubach F, Wells GA, Ravaud P, *et al.* Minimal clinically important difference, low disease activity state, and patient acceptable symptom state: methodological issues. *J Rheumatol* 2005;**32**:2025–9.

49 Concordance between important change and acceptable symptom state following knee arthroplasty: the role of baseline scores. *Osteoarthritis Cartilage* 2014;**22**:1107–10. doi:10.1016/j.joca.2014.06.006

50 Terluin B, Roos EM, Terwee CB, *et al.* Assessing baseline dependency of anchor-based minimal important change (MIC): don't stratify on the baseline score! *Qual Life Res Int J Qual Life Asp Treat Care Rehabil* 2021;**30**:2773–82. doi:10.1007/s11136-021-02886-2

51 de Vlam K, Ogdie A, Bushmakin AG, *et al.* Median time to pain improvement and the impact of baseline pain severity on pain response in patients with psoriatic arthritis treated with tofacitinib. *RMD Open* 2021;**7**:e001609. doi:10.1136/rmdopen-2021-001609

52 Dworkin RH, Turk DC, Peirce-Sandner S, *et al.* Assay sensitivity and study features in neuropathic pain trials: an ACTTION meta-analysis. *Neurology* 2013;**81**:67–75. doi:10.1212/WNL.0b013e318297ee69

53 Ziegler D, Pritchett YL, Wang F, *et al.* Impact of disease characteristics on the efficacy of duloxetine in diabetic peripheral neuropathic pain. *Diabetes Care* 2007;**30**:664–9. doi:10.2337/dc06-2009

54 Mercadante S, Adile C, Aielli F, *et al.* Personalized Pain Goals and Responses in Advanced Cancer Patients. *Pain Med Malden Mass* 2020;**21**:e215–21. doi:10.1093/pm/pnz254

55 Rodin R, Swami N, Pope A, *et al.* Impact of early palliative care according to baseline symptom severity: Secondary analysis of a cluster-randomized controlled trial in patients with advanced cancer. *Cancer Med* 2022;**11**:1869–78. doi:10.1002/cam4.4565

Supplementary Information 2. Standard Interventions for Vasomotor Symptoms

**Lifestyle Interventions**	• Environmental modifications such as dressing in layers, keeping the temperature of the room cool, using fans<br>• Dietary interventions like avoiding spicy food and alcohol<br>• Moderate-vigorous exercise for 150-180 minutes per week (ex. Swimming, cycling, jogging/running, weights)<br>• 90 minutes of hatha yoga weekly<br>• Relaxation practices-20 minutes once a day. Includes deep breathing exercises/box breathing, meditation, guided imagery, meditation app like Headspace®
**Complementary and Alternative Medicines**	**Botanicals:**<br>• Black Cohosh (Actea Racemosa)-40 mg OD<br>• Acteane®-1 tablet 2-4 times a day<br>• Evening Primrose oil-500 mg OD<br>**Other CAM Therapies:**<br>• Acupuncture-once weekly treatment, for example<br>• Melatonin-3-5 mg once nightly
**Pharmacologic**	**Antidepressants:**<br>• Venlafaxine (Effexor)-37.5 mg OD X 1 week then 75 mg OD<br>• Desvenlafaxine (Pristiq)-50 mg OD; increase daily dose in 25 to 50 mg increments every day to target dose of 100 mg OD<br>• Escitalopram (Ciprolex)-10 mg OD in the morning X 1 week then 20 mg OD<br>• Duloxetine 30 mg OD in the morning X 1 week then 60 mg OD<br>**Neuroleptics:**<br>• Gabapentin (Neurontin)-300 mg OD X 3 days, then 300 mg BID X 3 days, then 300 mg TID<br>• Pregabalin (Lyrica)-50 mg qHS; may increase at weekly intervals based on response and tolerability to 50 mg BID, and then up to 75 mg BID; may further increase up to 150 mg BID<br>**Other:**<br>• Clonidine-0.05 mg PO OD<br>• Oxybutynin-2.5-5 mg PO BID
**Breast Cancer Therapy Modification**	• Dose reduction (half dose every day or full dose q2days)<br>• Change of treatment to different drug (for example, tamoxifen to an AI)<br>• Interval treatment break (less than 6 weeks)

Supplementary Information 3: survey questions utilized in analysis

Baseline Questionnaire

1. **What is your current age?**
   Enter age____

2. **What was your menopausal status when you were diagnosed with breast cancer?**

   - Premenopause - you are continuing to have menstrual periods on a regular basis

   - Perimenopause - means "around menopause" and refers to the time during which your body makes the natural transition to menopause. Your menstrual cycles may lengthen or shorten, and you may also experience menopause-like symptoms, such as hot flashes, sleep problems and vaginal dryness.

   - Postmenopause - "Menopause" is defined as the final menstrual period and is usually confirmed when a woman has missed her period for more than 12 consecutive months (with no other obvious causes).

   - I don't know – for example women with an Intrauterine device (for example a Mirena IUD) may not have a period

3. **What treatments are you <u>currently</u> receiving for your breast cancer? (choose all that apply)**

- Chemotherapy
- Tamoxifen
- Aromatase inhibitors (anastrozole, letrozole, exemestane)
- Injections to stop your periods (e.g. leuprolide/Lupron, goserelin/Zoladex)
- Removal of ovaries
- Palbociclib (Ibrance), ribociclib (Kisqali), Abemaciclib (Verzenio)
- Trastuzumab (Herceptin) and/or TDM1 (Kadcyla)
- None of these treatments

The next section will ask you about *previous* treatments/interventions that you have tried to help manage your hot flashes (note- you may have taken these prior to your breast cancer diagnosis)

4. **Which lifestyle interventions have you tried to manage your hot flash symptoms? (choose all that apply)**

- Environmental medications such as dressing in layers, keeping the temperature of the room cool, using fans, etc.
- Dietary changes (eg. avoiding spicy food, avoiding alcohol/caffeine)
- Stopping smoking
- Exercise
- Yoga
- Relaxation practices/meditation
- I have not tried any of these interventions

5. Which complementary and alternative medicine interventions have you tried to manage your hot flash symptoms? (choose all that apply)

- Black cohosh (Actea Racemosa)
- Acteane (combination product)
- Evening primrose oil
- Soy
- Red clover
- Acupuncture
- Melatonin
- Vitamin E
- I have not tried any of these interventions
- None of these

6. Which anti-depressant prescription medications have you tried? (choose all that apply)

  - Escitalopram (Cipralex)
  - Sertraline (Zoloft)
  - Fluoxetine (Prozac)
  - Venlafaxine (Effexor)
  - Desvenlafaxine (Pristiq)
  - Duloxetine (Cymbalta)
  - I have not tried any of these interventions
  - None of these

7. What other prescription medications have you tried? (choose all that apply)

  - Gabapentin (Neurontin)
  - Pregabalin (lyrica)
  - Clonidine
  - Oxybutynin
  - I have not tried any of these interventions
  - None of these

8. What breast cancer therapy modifications have you tried? (choose all that apply)

- Dose reduction (ie- half dose prescribe every day, or full dose every 2nd day)
- Change of treatment to a different drug (eg. tamoxifen to anastrazole)
- Short treatment break

SUBJECTIVE ASSESSMENT OF HOT FLASHES

For the questions below, please circle your response from 1-10 based on how you feel this week.

1. To what extent do you view your hot flashes/nocturnal hot flashes as a problem?

1	2	3	4	5	6	7	8	9	10

Not a problem at all — Very much a problem

2. How distressed do you feel by your hot flashes?

1	2	3	4	5	6	7	8	9	10

Not a distressed at all — Very distressed

3. To what extent do your hot flashes interfere with your daily routine?

1	2	3	4	5	6	7	8	9	10

Not at all — Very much

4. How well are you coping with your hot flashes?

1	2	3	4	5	6	7	8	9	10

Not at all — Very much

5. How much control do you think you have over your hot flashes?

1	2	3	4	5	6	7	8	9	10

Not control at all — Very good control

6 Week Follow Up Questionnaire

1. Which intervention did you try as part of the study ? (check all that apply)

Lifestyle Modifications:

- Environmental modifications such as dressing in layers, keeping the temperature of the room cool, using fans
- Dietary interventions-Avoiding spicy food and/or alcohol
- Smoking cessation

- Exercise- 150-180 minutes per week, moderate-vigorous activity (eg. swimming, cycling, jogging/running)

- Yoga- 90 minutes of hatha yoga weekly

- Relaxation practices- 20 minutes once daily. Includes deep breathing exercises/box breathing, meditation, guided imagery. Example meditation app- Headspace®

Complementary and Alternative Therapies:

- Black Cohosh (Actea Racemosa)- 40 mg once daily
- Acteane®: 1 tablet 2 to 4 times daily (combination product)
- Evening Primrose oil- 500 mg once daily
- Acupuncture- Eg. once weekly treatments
- Melatonin- 3-5 mg once nightly

Prescription Medications:

- Venlafaxine (Effexor)- 37.5 mg once daily X 1 week then 75 mg once daily

- Desvenlafaxine (Pristiq)- 50 mg once daily; increase daily dose in 25 to 50 mg increments every day to target dose of 100 mg once daily

- Escitalopram (Cipralex)-10 mg once daily every morning X 1 week then 20 mg once daily

- Duloxetine 30 mg PO once daily every morning X 1 week then 60 mg once daily

- Gabapentin (Neurontin)-300 mg once daily X 3 days, then 300 mg twice daily X 3 days, then 300 mg 3 times daily

- Pregabalin (Lyrica)- 50 mg once daily at bedtime; may increase at weekly intervals based on response and tolerability to 50 mg twice daily, and then up to 75 mg twice daily; may further increase up to 150 mg twice daily

- Clonidine-0.05 mg twice daily

- Oxybutynin-2.5 mg twice daily or 5 mg twice daily

Changes to breast cancer therapy (eg. tamoxifen, anastrazole):

- Dose reduction (ie- half dose prescribed every day, or full dose every 2nd day)
- Change of treatment to different drug (eg tamoxifen → aromatase inhibitor)

- Short treatment break

None

- I have decided that I do not want/need an intervention at this time

2. Is this the <u>first</u> intervention that you have tried as part of the study?

- Yes
- No
- Not applicable

3. What date did you start the intervention, if applicable?
Insert date________
- Not applicable

<u>SUBJECTIVE ASSESSMENT OF HOT FLASHES</u>
For the questions below, please circle your response from 1-10 based on how you feel this week.

2. To what extent do you view your hot flashes/nocturnal hot flashes as a problem?

1 2 3 4 5 6 7 8 9 10

Not a problem at all — Very much a problem

3. How distressed do you feel by your hot flashes?

1 2 3 4 5 6 7 8 9 10

Not a distressed at all — Very distressed

4. To what extent do your hot flashes interfere with your daily routine?

1 2 3 4 5 6 7 8 9 10

Not at all — Very much

5. How well are you coping with your hot flashes?

1 2 3 4 5 6 7 8 9 10

Not at all — Very much

6. How much control do you think you have over your hot flashes?

1 2 3 4 5 6 7 8 9 10

Not control at all — Very good control

4. Please respond to this sentence

The intervention/treatment that I tried has adequately improved my hot flashes and/or night sweats.

Note- this question is applicable only if you started an intervention for your hot flashes

- Strongly agree
- Agree
- Neutral
- Disagree
- Strongly disagree
- Not applicable

Supplementary Information 4. Variables included in regression tree models for Hot Flush Rating Score, with degree of missingness reported

Variable	Description	Classification	n (%)
**Time**	Time in days between baseline and follow- up completion	Time > 56 days = 1<br>Time ≤ 56 days = 0	41 (39%)<br>63(61%)
**Intervention**	Intervention for VMS	Control (No intervention) = 0<br>Lifestyle = 1<br>CAM = 2<br>Prescription = 3<br>Endocrine therapy mod = 4	31 (30%)<br>27 (26%)<br>25 (24%)<br>12 (12%)<br>9 (9%)
**Age**	Age at time of baseline completion	Age < 49 = 0,<br>Age 49-63 = 1,<br>Age > 63 = 2<br>**Missing****	31 (30%)<br>51 (49%)<br>16 (15%)<br>6(6%)
**Menopausal status**	Menopausal status at time of breast cancer diagnosis	Pre/perimenopausal = 1<br>Post-menopausal = 0<br>**Missing**	51(49%)<br>42(40%)<br>11 (11%)
**Endocrine therapy duration**	Duration of time in months on current endocrine therapy	≤ 12 months = 1<br>> 12 months = 0<br>**Missing**	44 (42%)<br>39 (38%)_<br>21 (20%)
**Duration of time on intervention**	Duration of time in days on intervention for VMS	Continuous variable<br><br>**Missing**	Median 50.0 days (IQR 42.0-60.0).<br><br>59 (57%)

**Not all questions were mandatory for survey completion

Supplementary Information 5. Comparison of Median HFRS Change Scores Across Intervention Categories

	Control	Lifestyle	CAM	Prescription	Endocrine Modification
**Unadjusted Change**	-1.00 (-2.00, -0.33)	-0.67 (-1.17, 0.50)	-1.33 (-2.67, -0.34)	-0.33 (-1.67, 0.17)	-0.84 (-1.75, -0.67)
**Baseline Adjusted Change***	-0.68 (-1.97, 0.00)	-0.31 (-1.06, 0.00)	-1.33 (-2.63, -0.12)	-0.33 (-1.64, 0.03)	-0.61 (-1.75, -0.24)
**Relative Change**	-25% (-40%, -6%)	-14% (-32%, -10%)	-22% (-48, -7%)	-6% (-19%, 3%)	-19% (-0.33, -0.14)

***Values may differ from regression tree output as change scores based on intervention category only

Supplementary Information 6: Optimal Hyperparameters for HFRS Regression Tree

	**HFRS**
Minsplit	6
Minbucket	7
CP	0.08
Max depth	4
**Mean Squared Error**	**1.48**

Minsplit = minimum observation at node, Minbucket = minimal observation at terminal node,

CP = complexity parameter, Max depth= maximum number of splits in given tree

Supplementary Information 7: Further Analysis of Tree Terminal Nodes

Baseline Features of Select Terminal Nodes from the HFRS Regression Tree

	**Median Age (IQR)**	**Proportion of patients on Current Endocrine Therapy (n,%)**	**Proportion of patients on tamoxifen (n,%)**	**Duration of time on endocrine therapy (n,%)**
**Subgroup**				
**Post-menopausal and LS/control interventions** (median change -0.32)	61.0 (7.0, 62.0)	23 (89)	18 (69)	5.0 (3.80, 14.40)
**Pre menopausal lifestyle** (median change -0.08)	45.5 (43.0, 49.3)	10 (91)	9 (82)	8.8 (4.8, 22)
**Pre-menopausal control** (median change -0.84)	47.6 (44.5, 51.5)	15 (79)	10 (53)	18.5(7.35, 27.1)

Supplementary Information 8: TRIPOD Checklist

Section/Topic		Checklist Item	Section
**Title and abstract**			
Title	1	Identify the study as developing and/or validating a multivariable prediction model, the target population, and the outcome to be predicted.	Page 1
Abstract	2	Provide a summary of objectives, study design, setting, participants, sample size, predictors, outcome, statistical analysis, results, and conclusions.	Page 2
**Introduction**			
Background and objectives	3a	Explain the medical context (including whether diagnostic or prognostic) and rationale for developing or validating the multivariable prediction model, including references to existing models.	Section 1
	3b	Specify the objectives, including whether the study describes the development or validation of the model or both.	Section 1, paragraph 4
**Methods**			
Source of data	4a	Describe the study design or source of data (e.g., randomized trial, cohort, or registry data), separately for the development and validation data sets, if applicable.	Section 2.1
	4b	Specify the key study dates, including start of accrual; end of accrual; and, if applicable, end of follow-up.	Section 2.1
Participants	5a	Specify key elements of the study setting (e.g., primary care, secondary care, general population) including number and location of centres.	Section 2.1
	5b	Describe eligibility criteria for participants.	Section 2.1
	5c	Give details of treatments received, if relevant.	Section 2.1, paragraph 2
Outcome	6a	Clearly define the outcome that is predicted by the prediction model, including how and when assessed.	Section 2.2
	6b	Report any actions to blind assessment of the outcome to be predicted.	NA
Predictors	7a	Clearly define all predictors used in developing or validating the multivariable prediction model, including how and when they were measured.	Section 2.5, paragraph 5
	7b	Report any actions to blind assessment of predictors for the outcome and other predictors.	NA
Sample size	8	Explain how the study size was arrived at.	NA
Missing data	9	Describe how missing data were handled (e.g., complete-case analysis, single imputation, multiple imputation) with details of any imputation method.	Section 2.5, paragraph 1
Statistical analysis methods	10a	Describe how predictors were handled in the analyses.	Section 2.5, paragraph 4-6
	10b	Specify type of model, all model-building procedures (including any predictor selection), and method for internal validation.	Section 2.5, paragraph 7
	10d	Specify all measures used to assess model performance and, if relevant, to compare multiple models.	Section 2.5, paragraph 7
Risk groups	11	Provide details on how risk groups were created, if done.	NA
**Results**			
Participants	13a	Describe the flow of participants through the study, including the number of participants with and without the outcome and, if applicable, a summary of the follow-up time. A diagram may be helpful.	Section 3.1, 3.2
	13b	Describe the characteristics of the participants (basic demographics, clinical features, available predictors), including the number of participants with missing data for predictors and outcome.	Section 3.2, Suppl. File 4
Model development	14a	Specify the number of participants and outcome events in each analysis.	Section 3.3
	14b	If done, report the unadjusted association between each candidate predictor and outcome.	Suppl. R code
Model specification	15a	Present the full prediction model to allow predictions for individuals (i.e., all regression coefficients, and model intercept or baseline survival at a given time point).	Section 2.5, 3.3, Suppl.

			file 6, Suppl. R code
	15b	Explain how to the use the prediction model.	Section 3.4
Model performance	16	Report performance measures (with CIs) for the prediction model.	Section 3.4, Fig 3
**Discussion**			
Limitations	18	Discuss any limitations of the study (such as nonrepresentative sample, few events per predictor, missing data).	Section 4.3
Interpretation	19b	Give an overall interpretation of the results, considering objectives, limitations, and results from similar studies, and other relevant evidence.	Section 4.1
Implications	20	Discuss the potential clinical use of the model and implications for future research.	Section 4.2
**Other information**			
Supplementary information	21	Provide information about the availability of supplementary resources, such as study protocol, Web calculator, and data sets.	"Availability of Data"
Funding	22	Give the source of funding and the role of the funders for the present study.	"Funding"

Supplementary Information 9: Consolidated reporting guidelines for prognostic and diagnostic machine learning modeling studies (JMIR AI Checklist)

The following is the reporting checklist for ML modeling studies. A response should indicate whether the particular item is documented in the study. If the response to an item is Y then the location in the article should be provided (e.g., section number), and if the response is N or NA then some reasoning should be provided.

#	Item	Y	N	NA	Location / Reasoning
**Study Details**					
1.1	*The medical/clinical task of interest*	Y			Section 1
1.2	*The research question*	Y			Section 1, paragraph 4
1.3	*Current medical/clinical practice*	Y			Section 1
1.4	*The known predictors and confounders to what is being predicted / diagnosed*	Y			Section 1, paragraph 2
1.5	*The overall study design*	Y			Section 2
1.6	*The medical institutional setting(s)*	Y			Section 2.1, paragraph 1
1.7	*The target patient population*	Y			Section 2.1, paragraph 1
1.8	*The intended use of the ML model*	Y			Section 1,
1.9	*Existing model performance benchmarks for this task*			NA	Development of novel change score
1.10	*Ethical and other regulatory approvals obtained*	Y			“Ethics” section
**The Data**					
2.1	*Inclusion / exclusion criteria for the patient cohort*	Y			Section 2.1
2.2	*Methods of data collection*	Y			Section 2.1-2.3
2.3	*Bias introduced due to the method of data collection used*	Y			Section 4.3
2.4	*Data characteristics*	Y			Section 3.1, Table 1
2.5	*Methods of data transformations and preprocessing applied*	Y			Section 2.4
2.6	*Known quality issues with the data*	Y			Section 4.3
2.7	*Sample size calculation*		N		Exploratory analysis utilizing a novel method of assessing change scores

2.8	*Data Availability*	Y			"Availability of Data and Materials"
**Methodology**					
3.1	*Strategies for handling missing data*	Y			Section 2.5, Suppl. File 4, Section 2.5 paragraph 6
3.2	*Strategies for addressing class imbalance*			NA	Study intended to capture heterogeneity in intervention preferences
3.3	*Strategies for reducing dimensionality of data*			NA	Small low dimension dataset utilized
3.4	*Strategies for handling outliers*		N		All data fulfilling eligibility criteria analyzed
3.5	*Strategies for data augmentation*			NA	Outside of scope of study design
3.6	*Strategies for model pre-training*			NA	Outside of scope of study design
3.7	*The rationale for selecting the machine learning algorithm*	Y			Section 2.5, paragraph 3
3.8	*The method of evaluating model performance during training*	Y			Section 2.5, paragraph 7
3.9	*The method used for hyperparameter tuning*	Y			Section 2.5, paragraph 7
3.10	*Model's output adjustments*			NA	Outside of scope of study design
**Evaluation**					
4.1	*Performance metrics used to evaluate the model*	Y			Section 2.5, paragraph 7
4.2	*The cost or consequence of errors*			NA	Outside scope of study design
4.3	*The results of internal validation*	Y			Section 3.4
4.4	*The final model hyperparameters*	Y			Suppl. File 6
4.5	*Model evaluation on an external dataset*			NA	Data not available
4.6	*Characteristics relevant for detecting data shift and drift*			NA	Outside of scope of study design
**Explainability and Transparency**					
5.1	*The most important features and how they relate to the outcome(s)*		N		Outside of scope of study design
5.2	*Plausibility of model outputs*	Y			Section 4.1
5.3	*Interpretation of model's results by an end-user*	Y			Section 4.1, 4.2
